# Prevalence and imaging characteristics of cerebral small vessel disease in a Colombian population aged 40 years and older

**DOI:** 10.1093/braincomms/fcae057

**Published:** 2024-03-16

**Authors:** Laura Rodriguez, Ana T Araujo, Daniela D Vera, Adriana Rodríguez Gelvez, Paul A Camacho, Daniel E Mantilla, Juan C Mantilla

**Affiliations:** Radiology Department, Fundación Oftalmológica de Santander—Clínica Ardila Lülle, Floridablanca 681008, Colombia; Radiology Department, Fundación Oftalmológica de Santander—Clínica Ardila Lülle, Floridablanca 681008, Colombia; Radiology Department, Fundación Oftalmológica de Santander—Clínica Ardila Lülle, Floridablanca 681008, Colombia; Radiology Department, Fundación Oftalmológica de Santander—Clínica Ardila Lülle, Floridablanca 681008, Colombia; Research Group Fundación Oftalmológica de Santander—Clínica Ardila Lülle, Floridablanca 681008, Colombia; Radiology Department, Fundación Oftalmológica de Santander—Clínica Ardila Lülle, Floridablanca 681008, Colombia; Interventional Radiology Department, Fundación Oftalmológica de Santander—Clínica Ardila Lülle, Floridablanca 681008, Colombia; Radiology Department, Fundación Oftalmológica de Santander—Clínica Ardila Lülle, Floridablanca 681008, Colombia

**Keywords:** small vessel disease, brain aging, prevalence, MRI

## Abstract

Cerebral small vessel disease is a major contributor to both brain aging and cognitive decline. This study aimed to determine the prevalence of cerebral small vessel disease in a Colombian population over 40 years of age who attended a Radiology and Diagnostic Imaging service for brain MRI between October 2018 and March 2019. This was an observational, cross-sectional and analytical study of 710 adult patients over 40 years of age who attended the Radiology and Diagnostic Imaging service for a brain MRI. The analysed data were obtained from an anonymized database of the service. We studied 710 MRI scans of patients aged between 40 and 104 years. The most frequent risk factor was hypertension (36.2%). Brain abnormalities associated with cerebral small vessel disease, such as white matter hyperintensities, were seen in 56.20% of the population, and brain atrophy was observed in 12.96%. Brain disease prevalence increased with age (23.18% for those aged 55 years, 54.49% for those aged 55–64 years, 69.8% for those aged 65–74 years and 90.53% for those older than 75 years). The prevalence of cerebral small vessel disease in our population was similar to that reported in the world literature, as were the prevalence of the evaluated cardiovascular risk factors. Additionally, we identified an association between hypertension and advanced age with cerebral small vessel disease, with white matter hyperintensities being the most characteristic finding.

## Teaser

Rodriguez *et al*. investigated cerebral small vessel disease prevalence in adults over 40 years old via brain MRI. Hypertension was common (36.2%), and 56.20% had white matter hyperintensities. Prevalence increased with age (90.53% in >75). Findings align with global literature, highlighting hypertension, age and white matter hyperintensities’ significance.

## Graphical Abstract

**Graphical Abstract:**
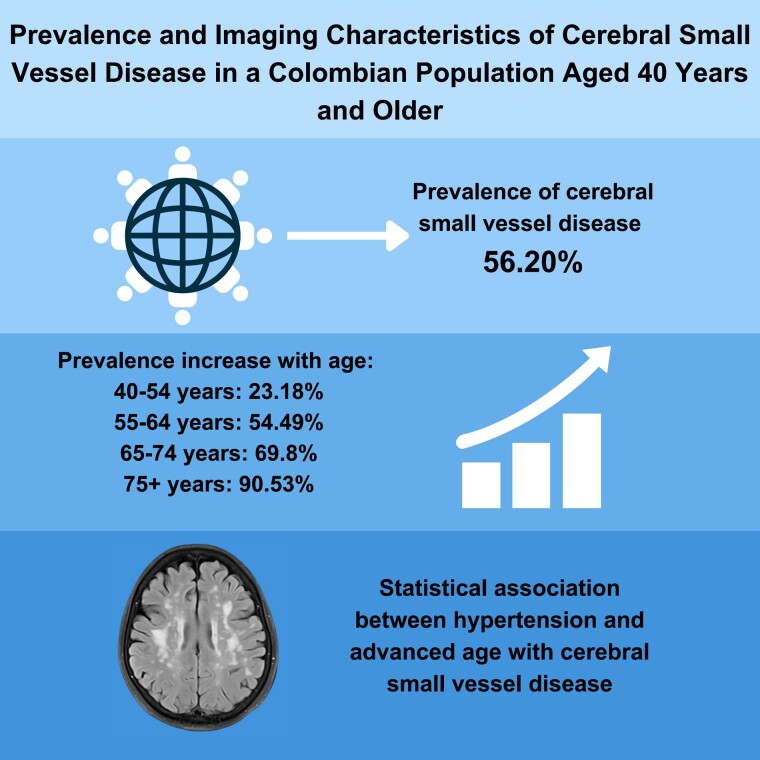

## Introduction

Cerebral small vessel disease (CSVD) presents a complex challenge in the realm of neurology. CSVD is a prominent contributor to both cognitive decline and cerebrovascular events, frequently characterized by cerebral white matter hyperintensities and lacunes, often detectable via MRI studies.^1^ CSVD has been identified as a leading vascular cause of dementia and accounts for approximately one-fifth of all stroke cases worldwide.^1^

Traditional MRI examinations have categorized CSVD into distinct entities, encompassing recent small subcortical infarcts, white matter hyperintensities, lacunes, perivascular spaces, cerebral microbleeds and brain atrophy, emphasizing its diverse manifestations.^2^

Although these changes were previously considered benign in older patients, recent international studies have shed light on their clinical significance. These alterations are no longer dismissed but recognized as markers of CSVD, closely linked to various morbidities, such as cognitive impairment, motor dysfunction, urinary issues and mood disorders.^1^ Furthermore, the implications of CSVD are substantial, not only affecting individual health but also carrying economic consequences. Studies from the USA suggest that reducing CSVD prevalence by as little as 20% could significantly decrease the lifetime incidence of dementia and lead to substantial cost savings.^1,3^

Currently, there are no studies in our population that evaluate the prevalence or imaging characteristics of cerebral small vessel disease, nor are there studies that investigate the relationship between cerebral small vessel disease and cognitive impairment. International studies in populations with different characteristics than ours cannot be extrapolated to our population. Therefore, it is important to study these alterations in our population to develop and implement preventive strategies that reduce disability in the elderly population and to establish criteria to identify high-risk patients early.

## Methodology

### Study design and participants

This cross-sectional observational study included adults aged 40 years and older who underwent a brain MRI at the Radiology and Diagnostic Imaging service. The study received approval from the Foscal Research Ethics Committee (Number 31, 07/09/2018), and all participants or their legal representatives provided written informed consent before enrolment in the study. Foscal and Foscal Internacional Clinic is a tertiary centre located in Floridablanca, Bucaramanga Metropolitan Area, Santander, Colombia, with ∼50 000 patients discharged from inpatient services annually. Participants were selected among patients who received a diagnostic brain MRI between October 2018 and March 2019. Exclusion criteria encompassed individuals with existing diagnoses of major neurocognitive disorder/dementia of any aetiology, white matter neurological diseases, intracranial malignancies or sequelae of severe head trauma. The reasons for undergoing brain MRI scans varied and were based on the clinical indications provided by the referring physicians.

### Data collection

The following information was registered by centralized well-trained interviewers: sociodemographic data, including age at diagnostic brain, educational level, socioeconomic condition, medical history, vascular risk factors, personal and family risk factors and access to health services were collected and kept masked for the radiology service and diagnostic images to prevent information bias.

Ischaemic lesions refer to specific regions of brain tissue that have experienced diminished blood flow, often due to the blockage or narrowing of small blood vessels. These lesions manifest distinct signal intensity patterns on various MRI sequences. On T_1_-weighted MRI sequences, ischaemic lesions typically appear hypointense, indicating chronic infarcts with long-standing tissue changes. In contrast, on T_2_-weighted and FLAIR (fluid-attenuated inversion recovery) MRI sequences, these lesions present as hyperintense areas, suggesting more recent or acute infarcts with altered tissue water content. Diffusion-weighted MRI, sensitive to restricted water diffusion in damaged tissue, reveals ischaemic lesions as hyperintensities, reflecting limited water mobility. Apparent diffusion coefficient maps, derived from diffusion-weighted MRI, complement this by quantifying the degree of water diffusion restriction, where ischaemic lesions typically exhibit hypointense regions.

### MRI acquisition

In the 1.5-tesla GE Signa Explorer and Toshiba Vantage equipment, with a matrix size of 256 × 256 cm², the following sequences are performed with a field of view of 24 and 5-mm slice thickness: sagittal T1 [repetition time (TR): 2000 ms and echo time (TE): 20 ms], axial T1 (TR of 2000 ms and a TE of 20 ms), axial T2 (TR of 4000 ms and TE of 100 ms), axial FLAIR [TR of 9000 ms, TE of 120 ms and an inversion time (TI) of 2200 ms], coronal T2 (TR of 4000 ms and TE of 100 ms), T2* magnetic susceptibility (TR of 600 ms and TE of 15 ms) and diffusion imaging utilized *b*-values of 0 and 1000 s/mm², with 12 diffusion directions, a TR of 4000 ms, and a TE of 100 ms.

### Quantification of CSVD

High-resolution MRI scans of the brain were meticulously reviewed by trained neuroradiologists to identify and characterize CSVD-related abnormalities, including white matter hyperintensities (WMH), lacune, perivascular spaces and cerebral microbleeds, based on visual criteria and clinical judgment. The Fazekas classification scale was employed to assess the severity and extent of CSVD-related lesions, particularly WMH. This widely-recognized classification system categorizes lesions into different grades based on their size, confluence and location, providing a comprehensive characterization of CSVD burden. Stringent quality control measures were implemented to ensure the accuracy and consistency of the visual assessments. Radiologists conducted visual inspections and verified adherence to the Fazekas classification criteria, maintaining precision in the quantification process.

### Statistical analysis

Qualitative variables were presented as counts and proportions with 95% confidence intervals (CIs), while quantitative variables were expressed as mean ± SD or median (Interquartile range) values, depending on the normality of the distribution evaluated by the Shapiro–Francia test. The participants were divided into two groups according to the absence and presence of CSVD; statistical comparison of the presence of cerebral small vessel disease with the chi-square or Fisher’s exact test for categorical variables and with Student’s *t* or Mann–Whitney tests for continuous variables, as appropriate. Association of cerebral ischaemia and the presence of WMH was determined using the chi-square test and binomial regression. Association between cerebral small vessel disease with sex, age, arterial hypertension, mellitus diabetes and smoking, estimated as binary-dependent variables, including small vessel brain disease. Common and adjusted prevalence ratios with their 95% CIs were calculated based on CSVD. To dismiss the underlying confounding bias in a cross-sectional observational study, we adjusted for potential covariates in the two models. In model 1, we did not adjust for any covariates and in model 2, we adjusted for sex, age, arterial hypertension, mellitus diabetes and smoking. All analyses were conducted with Stata 11.2 SE. All *P*-values were two sided with *P* < 0.05 considered statistically significant.

## Results

The study included the analysis of 710 brain MRIs, with 459 (64.65%) from female patients and 251 (35.35%) from male patients. The age range of the patients was between 40 and 104 years, with a mean age of 65.96 years. The most common cardiovascular risk factor observed was arterial hypertension, present in 36.2% of patients, followed by diabetes mellitus (12.96%) and smoking (6.90%). The primary reason for brain MRI scans was ‘Headache’ that was reported in 175 (24.64%) participants (Table 1).

**Table 1:** General characteristics of the population

Characteristics	*n* = 710 (%)
Gender	
Female	459 (64.65)
Male	251 (35.35)
Age	
<55 years	151 (21.27)
55–64 years	167 (23.52)
65–74 years	202 (28.45)
≥75 years	190 (26.76)
Arterial hypertension	257 (36.2)
Diabetes mellitus type 2	92 (12.96)
Smoking	49 (6.90)
Reasons for brain MRI scans	
Headache	175 (24.64)
Cognitive disorders	156 (21.97)
Vestibulocochlear symptoms	97 (13.66)
Vasculopathy	62 (8.73)
Cranial neuropathies	48 (6.76)
Movement disorders	31 (4.36)
Seizures	30 (4.22)
Other clinical concerns	30 (4.22)
Alterations in consciousness	23 (3.23)
Visual deficits	19 (2.67)
Gait disturbances	16 (2.25)
Hemiparesis	12 (1.69)
Postoperative follow-up	11 (1.54)

MRI, magnetic resonance imaging.

Regarding the distribution of brain abnormalities on MRI associated with cerebral small vessel disease, WMH were the most frequently observed (56.20%), followed by brain atrophy (12.96%), ischaemic lesions (7.75%), lacune (6.62%), perivascular spaces (6.34%) and cerebral microbleeds (4.65%) (Table 2).

**Table 2:** Distribution of small vessel brain disease

	Presence *n* (%)	Absence *n* (%)
White matter hyperintensities	399 (56.20)	311 (43.8)
Brain atrophy	92 (12.96)	618 (92.25)
Ischaemic lesion	55 (7.75)	655 (92.25)
Lacunes	47 (6.62)	663 (93.38)
Perivascular spaces	45 (6.34)	665 (93.66)
Cerebral microbleeds	33 (4.65)	677 (95.35)

The prevalence of cerebral small vessel disease increased with age, with 23.18% of patients under 55 years old presenting the disease, 54.49% in the 55–64 age group, 69.8% in the 65–74 age group and 90.53% in those over 75 years old (Table 3).

**Table 3:** Prevalence of small vessel brain disease by age group

Age	*n* (%)	95% CI	*P*-value
<55 years	35 (23.18)	0.16–0.29	<0.001
55–64 years	91 (54.49)	0.46–0.90	<0.001
65–74 years	141 (69.8)	0.63–0.76	<0.001
≥75 years	172 (90.53)	0.86–0.94	<0.001

**Figure 1:**
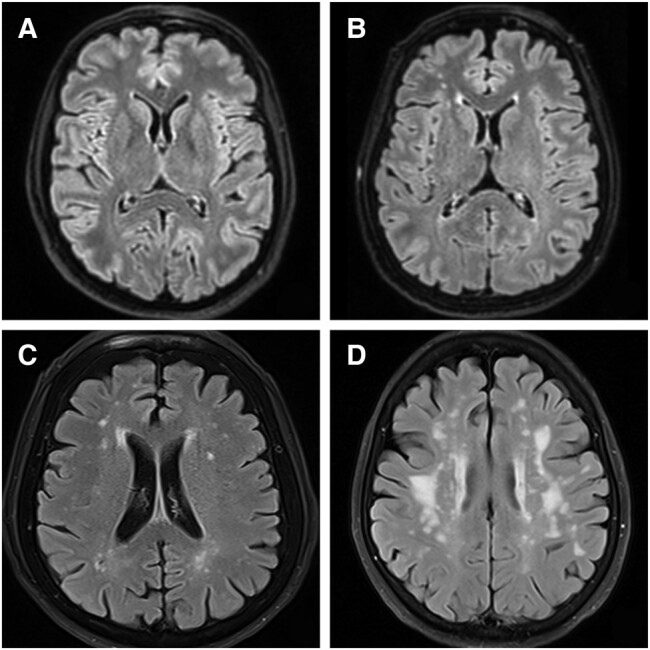
**Brain MRI showing different Fazekas classification types.** (**A**) Fazekas 0: no white matter hyperintensities. (**B**) Fazekas 1: punctate hyperintensities. (**C**) Fazekas 2: early confluent hyperintensities. (**D**) Fazekas 3: confluent hyperintensities with early ventricular involvement.

The presence of a degree of WMH (56.2%), according to the Fazekas classification, was more prevalent in punctate lesions or Fazekas 1 (Table 4). According to the distribution of Fazekas by age, it was determined that 82.7% of patients under 55 years old did not show any WMH and were categorized as Fazekas 0. Within this age range, the second classification with the highest prevalence was isolated lesions/Fazekas 1. Among the population aged 55–64 years, the absence of lesions predominated (50.90%), and punctate lesions (40.12%) were the second most prevalent. The presence and severity of lesions increased progressively in patients aged 65–74 years, with punctate lesions being present in 43.56% of patients. In patients over 75 years old, the majority had WMH (84.74%), and punctate lesions were the most frequently identified (36.32%), followed by partially confluent lesions (27.89%) and confluent lesions categorized as Fazekas 3 (20.53%), which was the classification with the highest prevalence in this age group (Table 5, Fig. 1).

**Table 4:** Distribution of hyperintensities of subcortical brain according to Fazekas classification

Fazekas		*n* (%)	95% CI	*P*-value
0	Absence of injury	311 (43.8)	95% 0.40–0.47	0.843
1	Punctate lesions	249 (35.07)	95% 0.31–0.38	0.843
2	Partially confluent lesions	99 (13.94)	95% 0.11–0.16	0.843
3	Confluent lesions	51 (7.18)	95% 0.05–0.09	0.843

**Table 5:** Fazekas distribution according to age category

Age	Fazekas 0	Fazekas 1	Fazekas 2	Fazekas 3	*P*-value
<55 years	125 (82.78)	25 (16.56)	1 (0.66)	0 (0.0)	<0.001
55–64 years	85 (50.90)	67 (40.12)	11 (6.59)	4 (2.40)	<0.001
65–74 years	72 (35.64)	88 (43.56)	34 (16.83)	8 (3.96)	<0.001
>75 years	29 (15.26)	69 (36.32)	53 (27.89)	39 (20.53)	<0.001

Non-lacunar infarcts refer to cerebral infarctions that are not characterized by small, deep lesions often associated with lacunes. Instead, non-lacunar infarcts typically involve larger, more extensive areas of brain tissue and are often associated with other cerebrovascular conditions. The presence of non-lacunar infarcts, regardless of their time of evolution, was associated with the presence of WMH, with 61.81% of patients classified in Fazekas Groups 1 (36.36%) and 2 (25.45%) (Table 6).

**Table 6:** Non-lacunar infarcts and Fazekas

Infarcts	Fazekas 0	Fazekas 1	Fazekas 2	Fazekas 3	*P*-value
No	296 (45.19%)	229 (34.96%)	85 (12.99%)	45 (6.87%)	0.034
Yes	15 (27.27%)	20 (36.36%)	14 (25.45%)	6 (10.91%)	0.034

The univariate analysis found an association between the different cardiovascular risk factors studied and cerebral small vessel disease. However, in the multivariate analysis, only age and arterial hypertension were found to have statistical significance (Table 7).

**Table 7:** Univariate and multivariate analyses based on small vessel brain lesions

	RP crude	*P*-value	Corrected RP	*P*-value
Gender				0.737
Female	Reference			
Male	1.02 [95% CI 0.89 a 1.16]	0.757	0.98 [95% CI 0.89 a 1.08]	
Age				<0.001
<55 years	Reference			
55–64 years	2.8 [95% CI 1.94 a 4.17]	<0.001	2.78 [95% CI 1.90 a 4.08]	
65–74 years	3.73 [95% CI 2.5 a 5.38]		3.62 [95% CI 2.51 a 5.23]	
>75 years	4.9 [95% CI 3.45 a 7.01]		4.62 [95% CI 3.22 a 6.64]	
Arterial hypertension	1.5 [95% CI 1.37 a 1.75]	<0.001	1.14 [95% CI 1.02 a 1.27]	0.014
DM	1.3 [95% CI 1.17 a 1.56]	<0.001	0.97 [95% CI 0.85 a 1.10]	0.671
Smoking	1.29 [95% CI 1.07 a 1.56]	0.007	0.96 [95% CI 0.81 a 1.13]	0.654

DM, diabetes mellitus; RP, Prevalence Ratio.

## Discussion

In our study, we found a significant association between the presence of cerebral small vessel disease and age and hypertension in the multivariate analysis, which is consistent with previous research. The Standards for Reporting Vascular Changes on Neuroimaging (STRIVE v1) study by Wardlaw *et al*.^4^ identified a spectrum of lesions that cerebral small vessel disease can include, such as WMH, small subcortical infarcts, lacunes, cerebral microbleeds and more. While Vermeer *et al*.^5^ reported a prevalence of WMH ranging from 8–28%, our study found them in 56.20% of the population, followed by brain atrophy (12.96%) and lacunes (7.75%). Despite being frequently ignored or undervalued in clinical practice, WMH have been linked to the development of cognitive impairment, affective disorders, motor disturbances and death, as well as symptomatic stroke, independent of the presence of other vascular risk factors, and the development of dementia.^3,6–9^

The clinical presentation of cerebral small vessel disease can vary depending on the location, extent and type of vascular lesion. Most symptoms are related to neurodegenerative effects secondary to cerebral small vessel disease, such as brain atrophy.^4^ In our study, the main reasons for consultation were headache, cognitive impairment in 24.64%, vestibular–cochlear symptoms in 21.97% and vascular pathology in 8.73%.

The Rotterdam Study, which included 1077 patients aged 60–90 years without dementia or stroke, concluded that the presence of cerebral small vessel disease on baseline MRI was associated with worse performance in neuropsychological tests and greater cognitive function decline. Several studies have reported a strong association between cerebral small vessel disease and cognitive impairment.^10^ For instance, a systematic review of eight studies showed that an increase in WMH is linked to greater global cognitive decline in high-risk populations. The LADIS study also found a higher risk of neurological decline in individuals with cerebral small vessel disease after 3 years of follow-up.^11^ The volume of WMH beyond what could be explained by age and other demographic characteristics was found to be responsible for a difference in cognitive performance in individuals aged 60 years and older.

The Silent Brain Infarcts and the Risk of Dementia and Cognitive Decline study followed 3697 person-years (with a mean of 3.6 years per person) and found that the presence of silent infarcts at baseline more than doubled the risk of dementia. Thalamic infarcts were associated with memory impairment, and non-thalamic infarcts were associated with language impairment.^5^

One limitation of this study is that it is a cross-sectional study designed to measure the prevalence of cerebral small vessel disease, which does not allow for the identification of causal associations. The design of this study requires that the prevalence of the event of interest be relatively high, and the sample size was calculated based on cerebral small vessel disease as a whole and not on each of its components. The sample size was insufficient to yield representative results for less prevalent findings. Additionally, the use of a convenience sample in our study may limit the generalizability of our findings. Recognizing the importance of broader context, it is noteworthy that a population-based sample would have been more appropriate for addressing the research question. The interpretation of the images was done by a single observer. Additionally, it’s important to acknowledge that our study lacked reader-independent volumetric assessment using a validated segmentation tool due to resource constraints at our centre.

## Conclusion

The prevalence of cerebral small vessel disease in our population was similar to that reported in the global literature, as were the prevalence of the evaluated cardiovascular risk factors. Additionally, we identified an association between hypertension and advanced age with cerebral small vessel disease, with WMH being the most characteristic finding.

## Data Availability

The dataset and anonymized patient information used in this study are available upon reasonable request. Researchers interested in accessing the data for further analysis or validation of findings can contact the corresponding author for assistance in obtaining the necessary approvals and access to the dataset.
